# Development and external validation of a predictive model for type 2 diabetic retinopathy

**DOI:** 10.1038/s41598-024-67533-5

**Published:** 2024-07-20

**Authors:** Yongsheng Li, Bin Hu, Lian Lu, Yongnan Li, Siqingaowa Caika, Zhixin Song, Gan Sen

**Affiliations:** 1 Department of Preventive Medicine, Medical College, Tarim University, Alar, 843300 China; 2 Department of Medical Engineering and Technology, Xinjiang Medical University, Ürümqi, 830011 China; 3 Nursing Department, Suzhou BenQ Hospital, Suzhou, 215163 China; 4 Nursing Department, First Affiliated Hospital of Xinjiang Medical University, Ürümqi, 830054 China

**Keywords:** Diabetes retinopathy, Prediction model, Nomogram, 25(OH)D3, Type 2 diabetes mellitus (T2DM), Diabetes complications, Diagnostic markers, Predictive markers, Machine learning, Predictive medicine, Statistical methods

## Abstract

Diabetes retinopathy (DR) is a critical clinical disease with that causes irreversible visual damage in adults, and may even lead to permanent blindness in serious cases. Early identification and treatment of DR is critical. Our aim was to train and externally validate a prediction nomogram for early prediction of DR. 2381 patients with type 2 diabetes mellitus (T2DM) were retrospective study from the First Affiliated Hospital of Xinjiang Medical University in Xinjiang, China, hospitalised between Jan 1, 2019 and Jun 30, 2022. 962 patients with T2DM from the Suzhou BenQ Hospital in Jiangsu, China hospitalised between Jul 1, 2020 to Jun 30, 2022 were considered for external validation. The least absolute shrinkage and selection operator (LASSO) and multivariate logistic regression was performed to identify independent predictors and establish a nomogram to predict the occurrence of DR. The performance of the nomogram was evaluated using a receiver operating characteristic curve (ROC), a calibration curve, and decision curve analysis (DCA). Neutrophil, 25-hydroxyvitamin D3 [25(OH)D3], Duration of T2DM, hemoglobin A1c (HbA1c), and Apolipoprotein A1 (ApoA1) were used to establish a nomogram model for predicting the risk of DR. In the development and external validation groups, the areas under the curve of the nomogram constructed from the above five factors were 0.834 (95%CI 0.820–0.849) and 0.851 (95%CI 0.829–0.874), respectively. The nomogram demonstrated excellent performance in the calibration curve and DCA. This research has developed and externally verified that the nomograph model shows a good predictive ability in assessing DR risk in people with type 2 diabetes. The application of this model will help clinicians to intervene early, thus effectively reducing the incidence rate and mortality of DR in the future, and has far-reaching significance in improving the long-term health prognosis of diabetes patients.

## Introduction

In recent decades, the prevalence of diabetes mellitus (DM) has risen significantly worldwide, becoming a progressively more serious public health problem^1^. According to the International Diabetes Federation^2^, China, India, and the United States are the three countries with the highest rates of diabetes in 2019. This trend is predicted to continue to rise from 2030 to 2045, with China and India facing a huge burden of the disease.

Diabetic retinopathy (DR), one of the most common chronic complications of diabetes, is also the leading cause of irreversible visual impairment in adults, and in severe cases, can even lead to permanent blindness^3^. Notably, patients with DR may not have obvious symptoms until the disease is advanced. It is estimated that about 30% of the approximately 463 million patients diagnosed with diabetes worldwide also suffer from diabetic retinopathy^4^, and in our previous study^5^, we found that the prevalence of DR in the Xinjiang region of China was as high as 42.26%, which is much higher than the global average. In addition, according to relevant guidelines, it is recommended that diabetic patients should undergo a comprehensive eye examination within 5 years of diagnosis or at the time of diagnosis^4,6^. However, current DR diagnostic methods mainly rely on the detection of clinical symptoms, and although advances in imaging technology have improved diagnostic accuracy, these methods are costly, labor-intensive, and time-consuming when performing large-scale DR screening^7^. On the other hand, China, as a developing country, suffers from a lack of capacity for early detection of diabetic retinopathy and lacks a national DR screening program^8^. There are various reasons for this problem, including the lack of effective screening tools, shortage of ophthalmic professionals, and insufficient multidisciplinary strategies from image capture to DR diagnosis^9^. Further, regional economic disparities and differences in lifestyle make it challenging to replicate a DR management approach in China. This is particularly problematic in settings with limited healthcare resources, especially in regions where DR screening rates are significantly lower than the national average, such as Xinjiang, China. Therefore, the development of a simple, intuitive, and personalized prediction model is essential to stop the progression of the disease and protect patients vision^10^.

Prior to this study, we have developed a prediction model for DR in Xinjiang^5^. However, this model is highly dependent on whether the patient also has diabetic peripheral neuropathy (DPN), which occupies an extremely important position in the model, which greatly increases the difficulty of generalizing the model. In another of our studies^11^, we found that 25 hydroxyvitamin D3 [25(OH)D3] is an important risk factor for DPN. Also, existing studies have shown a strong relationship between 25(OH)D3 and DR^12–14^. In addition, the viewpoint that ApoA1 is more stable compared to lipid indicators has been proposed, but so far, no research has constructed DR risk prediction models using 25(OH)D3 and ApoA1 as risk factors. Therefore, we plan to construct a novel DR risk prediction model by using 25(OH)D3 as a risk factor for the first time based on previous studies. This model is intended to provide a reference for clinicians in the early screening, diagnosis, and treatment of DR, as well as the possibility of prioritizing retinal screening for patients who are at risk for DR but are unable to undergo routine eye examinations.

## Methods

### Study design and population

This is a retrospective analysis. The study protocol was approved by the Ethics Committees of First Affiliated Hospital of Xinjiang Medical University and Suzhou BenQ Hospital with a waiver for informed consent. The research methods were carried out in accordance with relevant guidelines and regulations.

In the development group, the retrospective study collected data of 2381 patients pathologically diagnosed with T2DM who were first admitted to the First Affiliated Hospital of Xinjiang Medical University in Xinjiang, China, between Jan 1, 2019 and Jun 30, 2022. In the external validation group, 962 patients with T2DM were Suzhou BenQ Hospital in Jiangsu, China, from Jul 1, 2020 to Jun 30, 2022.

The diagnostic criteria for type 2 diabetes mellitus was based on the Guideline for the prevention and treatment of type 2 diabetes mellitus in China (2017 Edition)^15^. According to these guidelines^15^, at the end of the routine examination, all patients were injected with 5 ml of sodium fluorescein at a concentration of 100 g/L into the elbow vein for fundus fluorescence angiography. About 10 s after the injection, a picture was taken with a KOWA fundus fluorescence camera. Subsequently, the captured images were analyzed using computer image processing techniques. Finally, two ophthalmologists reviewed and analyzed the results and made a diagnosis of diabetic retinopathy.

DPN: (a) abnormal vibration perception, (b) abnormal temperature perception, (c) ankle reflex disappeared, (d) nylon filament examination, foot sensation decreased or disappeared, and (e) nerve conduction velocity (NCV). If someone has two or more of the above phenomena, he/she is diagnosed as having DPN.

Inclusion criteria were as follows: (a). all research participants were able to communicate independently, and (b). patients at least 18 years old. Exclusion criteria were as follows: (a). incomplete clinical data, (b). Severe hepatic and renal insufficiency, and (c). Combined familial fundus disease.

### Data collection

All baseline clinical characteristics, including gender, age, white blood cell, total cholesterol, high-density lipoprotein, low-density lipoprotein, direct bilirubin, indirect bilirubin, total bilirubin, aspartate aminotransferase, alanine aminotransferase, body mass index, systolic blood pressure, diastolic blood pressure, serum creatinine, hemoglobin A1c, glycosylated serum protein, apolipoprotein A1, apolipoprotein B, fasting blood glucose, triglyceride, blood urea nitrogen, 25-hydroxyvitamin D3, cystatin C, 2-h postprandial blood glucose,homocysteine, blood glucose, DR.

### Statistical analysis

Statistical analysis was performed using R software (version 4.2.1). The continuous variables with normal distribution and homogeneity of variance are expressed as means ± standard deviations and tested using an independent sample t-test. Categorical variables were reported as percentages and compared using the Chi-square test. Independent risk factors were selected using least absolute shrinkage and selection operator (LASSO) regression and multivariate logistic regression^16^. A nomogram for identifying DR occurrence was constructed using covariates selected by multivariate regression, and discriminatory ability was assessed by measuring the area under the receiver operating characteristic curve (ROC)^17^. The Hosmer–Lemeshow goodness of fit test was used to assess the model fit and efficacy of the risk model^18^. Decision curve analysis and clinical impact curves were used to assess the clinical applicability of the nomogram^19^. All statistical analyses were two-sided, and statistical significance was set at p < 0.05.

### Institutional review board statement

This study was conducted by the Declaration of Helsinki and was approved by the Ethics Committees of First Affiliated Hospital of Xinjiang Medical University (K202105-05) and Suzhou BenQ Hospital (SZMJYY2022102001) with a waiver for informed consent.

### Informed consent statement

Waiver for informed consent.

## Results

### Patient characteristics

A total of 3537 patients with T2DM were screened (2526 in the development group and 1011 in the external validation group, respectively), of which 194 were excluded due to incomplete medical records (145 in the development group and 49 in the external validation group, respectively), and 3343 patients were finally included.

The detailed baseline demographics and clinical characteristics of the patients in the two groups are listed in Table 1. For the development group, the clinical information a total of 2381 patients was obtained from First Affiliated Hospital of Xinjiang Medical University. The patients (1464 males and 917 females) had a mean (SD) age of 57.268 ± 12.128 years of which the prevalence of DR was 44.6%. For the external validation group, the clinical information a total of 962 patients was obtained from Suzhou BenQ Hospital. The patients (627 males and 335 females) had a mean (SD) age of 57.279 ± 12.710 years, of which the prevalence of DR was 36.4%.

**Table 1:** Baseline characteristics of all patients in the development group and external validation group.

Variables	Development group (n = 2381) Mean (SD)/N (%)	External validation group (n = 962) Mean (SD)/N (%)	P
DR (%)			< 0.001
No	1318 (55.4%)	621 (64.6%)	
Yes	1063 (44.6%)	341 (36.4%)	
Gender (%)			0.046
M	1464 (61.5%)	627 (65.2%)	
F	917 (38.5%)	335 (34.8%)	
Age (years)	57.268 (12.128)	57.279 (12.710)	0.981
WBC (*10^9^)	7.184 (2.534)	7.032 (2.377)	0.11
Neutrophil (*10^9^)	4.294 (2.110)	4.094 (1.979)	0.011
Eosinophil (*10^9^)	0.167 (0.160)	0.197 (0.243)	< 0.001
Lymphocyte (*10^9^)	2.179 (0.799)	2.134 (0.711)	0.134
Hemoglobin (g/L)	137.521 (19.221)	140.088 (18.516)	< 0.001
Platelet (*10^9^)	226.510 (66.408)	231.289 (69.334)	0.063
TC (mmol/L)	4.252 (1.105)	4.125 (1.120)	0.003
HDL (mmol/L)	1.096 (0.344)	1.102 (0.340)	0.649
LDL (mmol/L)	2.719 (0.886)	2.625 (0.903)	0.006
DB (U/L)	3.474 (2.004)	3.738 (2.055)	< 0.001
IB (U/L)	7.919 (4.535)	7.837 (4.994)	0.646
TB (U/L)	11.394 (5.553)	11.576 (6.194)	0.406
AST (U/L)	20.921 (13.054)	21.624 (15.943)	0.188
ALT (U/L)	25.193 (20.747)	27.401 (22.696)	0.007
BMI (kg/m^2^)	26.064 (3.801)	26.060 (3.680)	0.98
SBP (mmHg)	127.180 (16.108)	123.172 (17.958)	< 0.001
DBP (mmHg)	76.784 (9.773)	73.681 (10.667)	< 0.001
Duration of T2DM	8.424 (7.247)	8.027 (7.258)	0.152
Scr (μmol/L)	72.341 (30.847)	70.703 (30.127)	0.162
HbA1c (%)	8.781 (2.130)	8.839 (2.143)	0.476
GSP (%)	2.769 (0.682)	2.726 (0.747)	0.111
ApoA1 (g/L)	1.168 (0.247)	1.162 (0.264)	0.537
ApoB (g/L)	0.926 (0.283)	0.935 (0.295)	0.419
FBG (mmol/L)	8.746 (3.035)	8.812 (2.901)	0.565
TG (mmol/L)	2.333 (2.178)	2.327 (2.368)	0.938
BUN (mmol/L)	7.034 (16.196)	7.091 (16.574)	0.927
25(OH)D3 (ng/mL)	16.121 (7.372)	16.657(8.018)	0.064
Cys C (mg/L)	2.357 (0.842)	2.298 (0.801)	0.063
PBG (mmol/L)	18.047 (4.522)	17.874 (4.270)	0.310
Hcy (μmol/L)	12.891 (2.904)	13.109 (2.796)	0.047
BG (mmol/L)	12.576 (2.791)	11.871 (2.598)	0.648

DR: diabetic retinopathy; M: male; F: female; WBC, white blood cell; TC: total cholesterol; HDL: high-density lipoprotein; LDL: low-density lipoprotein; DB: direct bilirubin; IB: Indirect Bilirubin; TB: total bilirubin; AST: aspartate aminotransferase; ALT: alanine aminotransferase; BMI: Body mass index; SBP: Systolic blood pressure; DBP: diastolic blood pressure; Scr: serum creatinine; HbA1c: hemoglobin A1c; GSP: glycosylated serum protein; ApoA1: apolipoprotein A1; ApoB: apolipoprotein B; FBG: fasting blood glucose; TG: Triglyceride; BUN: blood urea nitrogen; 25(OH)D3: 25-hydroxyvitamin D3; Cys C: cystatin C; PBG: 2-h postprandial blood glucose; Hcy: homocysteine; BG: blood glucose.

### Screening for predictive factors

**Figure 1:**
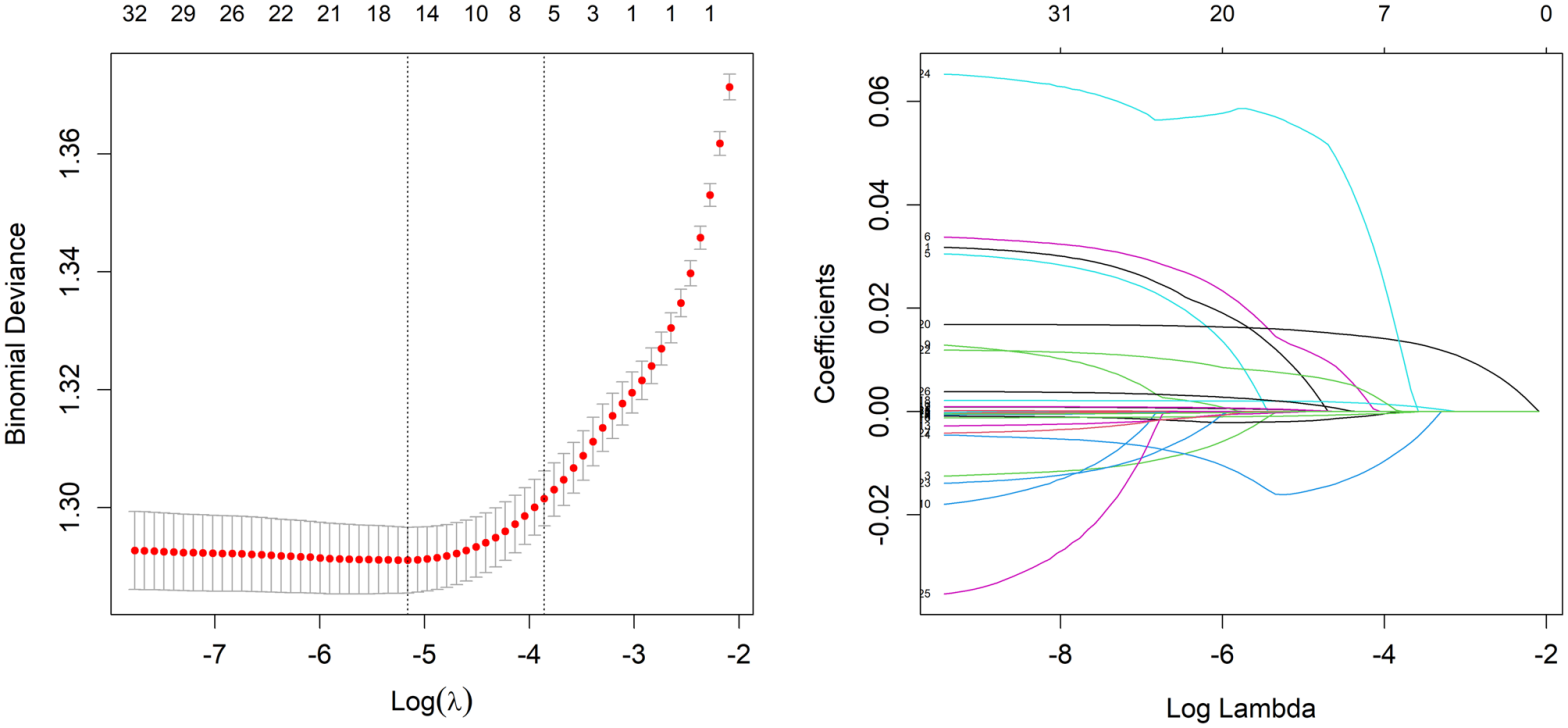
Demographic and clinical feature selection using the LASSO regression model.

We use the 2381 patients in the development group to choose features with nonzero coefficients in the LASSO regression model. The variables in the model were gradually lowered when the penalty coefficient was changed. Eventually, the tenfold cross-validation error was chosen as the minimum λ + 1 (lambda. 1se = 0.024) and optimal value of the model; at this point, we have 5 variables (Fig. 1). These potential predictors consisted of Neutrophil, 25(OH)D3, Duration of T2DM, HbA1c, and ApoA1. These five candidate variables were then analyzed using multivariate logistic regression. Ultimately, the five variables of Neutrophil (OR = 0.908, 95% CI (0.870, 0.945), p < 0.001); 25(OH)D3 (OR = 0.877, 95% CI (0.863, 0.891), p = 0.001); Duration of T2DM (OR = 1.167, 95% CI (1.134, 1.210), p < 0.001); HbA1c (OR = 1.172, 95% CI (1.094, 1.213), p < 0.001); and ApoA1 (OR = 1.806, 95% CI (1.311, 2.492), p < 0.001) were statistically significant and hence selected for the development of the prediction model (Table 2).

**Table 2:** Multivariate Logistic Regression analysis for risk factors of DR.

Variables	OR	95% CI	P
Neutrophil	0.908	(0.870, 0.945)	< 0.001
25(OH)D3	0.877	(0.863, 0.891)	0.001
Duration of T2DM	1.167	(1.134, 1.210)	< 0.001
HbA1c	1.172	(1.094, 1.213)	< 0.001
ApoA1	1.806	(1.311, 2.492)	< 0.001

25(OH)D3: 25-hydroxyvitamin D3; HbA1c: hemoglobin A1c; ApoA1: apolipoprotein A1.

### Risk prediction nomogram development

**Figure 2:**
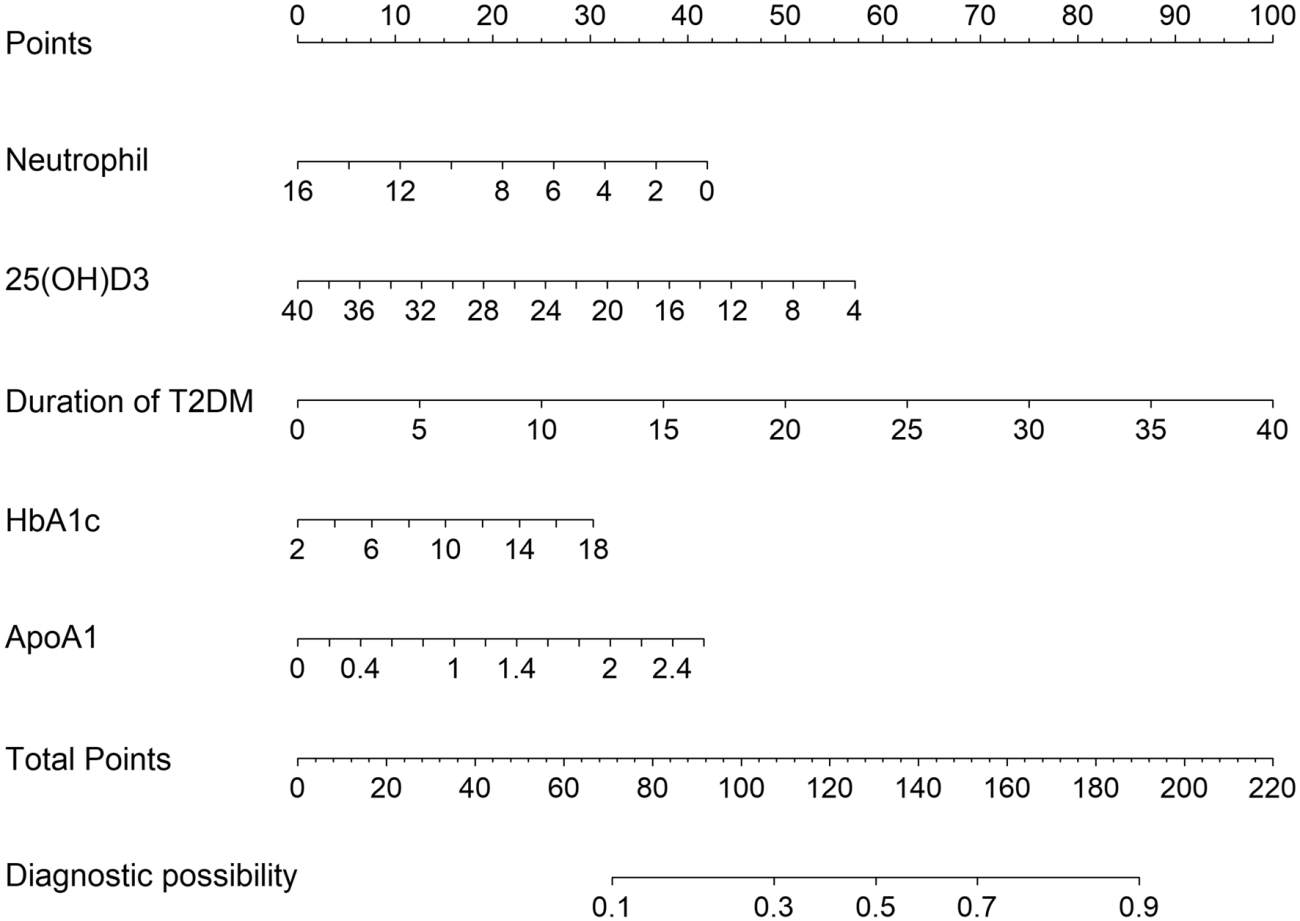
Nomogram for the perinatal prediction of DR. DR: diabetic retinopathy.

Based on the LASSO regression and multivariate logistic regression analysis, and clinical correlation results, five predictors from the development group were included in the DR risk prediction model and presented as a nomogram (Fig. 2). The nomogram illustrated that each predictor corresponded to a specific score ranging from 0 to 100. The total score is determined by adding the scores of each predictor and is located on the “Total Points” axis. The probability of DR corresponds to the bottom “Diagnostic possibility” axis of each patient. A higher score for a related factor in the nomogram indicates a higher risk of developing DR.

### Predictive accuracy and net benefit of the nomogram

**Figure 3:**
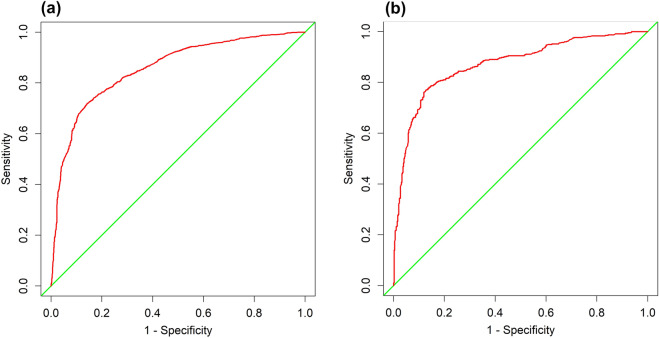
ROC curves. (**A**) Development group. (**B**) External validation group. ROC: receiver operating characteristic; AUC: area under the ROC curve.

**Figure 4:**
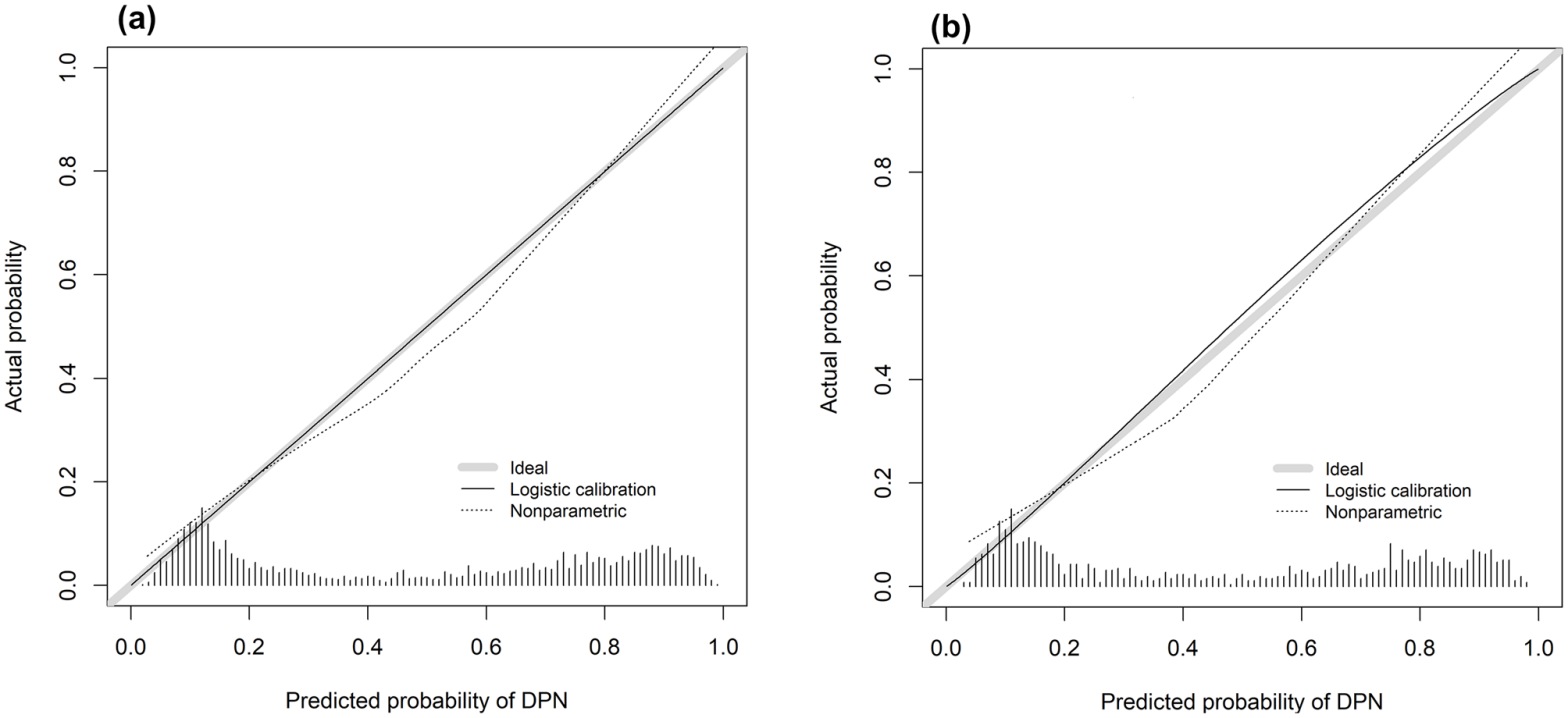
Calibration curve for predicting probability of DR. (**A**) Development group. (**B**) External validation group. DR: diabetic retinopathy.

For the development group, The area under the curve (AUC) was 0.834(95%CI 0.820–0.849) (Fig. 3A), and the calibration curve was close to the ideal diagonal line (Fig. 4A), Hosmer–Lemeshow test showed the model was in line with observed data (p > 0.05). In addition, 962 patients from Suzhou BenQ Hospital were used for the external validation to test the nomogram. The AUC was 0.851(95%CI 0.829–0.874) (Fig. 3B), reflecting a good accuracy of the nomogram. Meanwhile, the model had good consistency, and the calibration curve of the external validation group was also close to the ideal diagonal line (Fig. 4B), Hosmer–Lemeshow test showed the model was in line with observed data (p > 0.05) as well.

**Figure 5:**
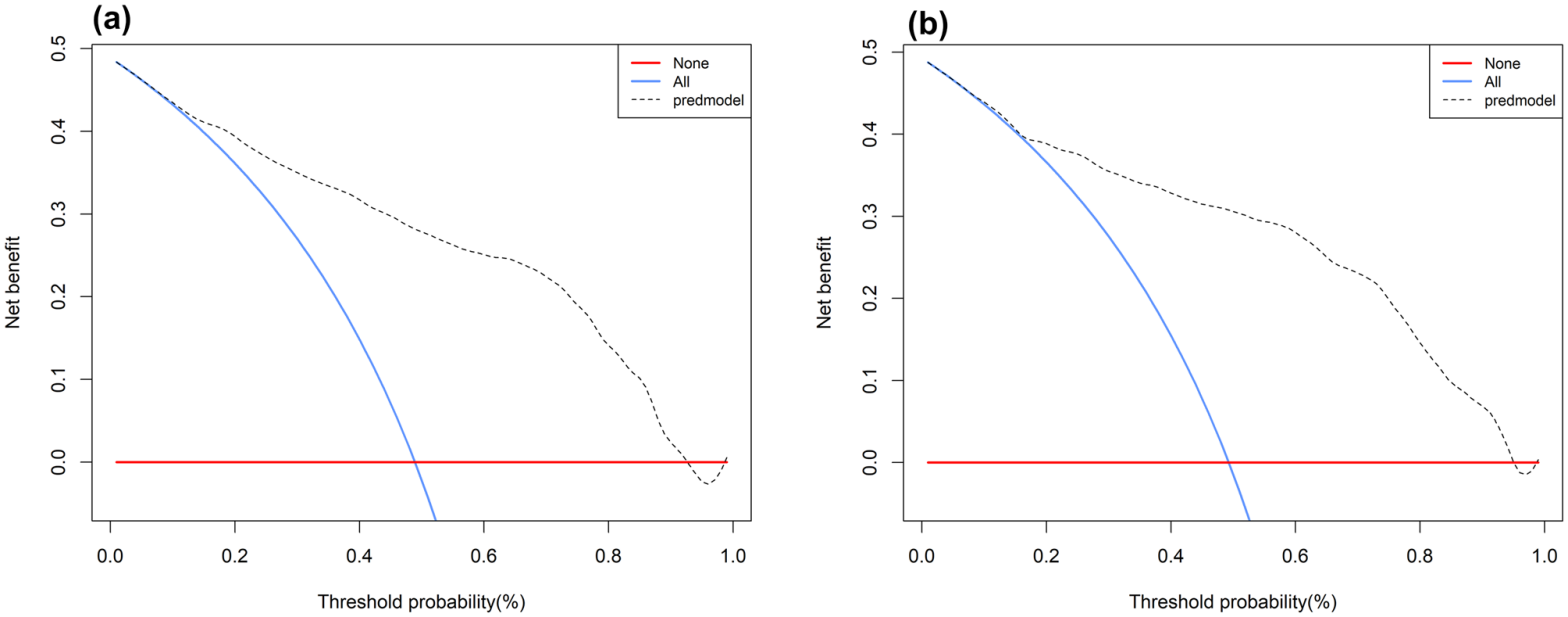
Decision curve analysis in prediction of DR. (**A**) Development group. (**B**) External validation group. DR: diabetic retinopathy.

The result of decision curve analysis for the nomogram is presented in Fig. 5 (Net benefits for different threshold probabilities were showed in Table S1, Supplementary Material). In the development and external validation groups, the decision curve showed that if the threshold probability of a patient were in the range of 0–0.92 and 0–0.95, respectively, which using the model achieved more net benefits than the "full treatment" or "no treatment" strategy. There was a broad spectrum of alternative threshold probability, suggesting that the model was a good assessment tool.

## Discussion

Building on our previous study, the present study innovatively developed a DR risk prediction model that integrates five key variables: Neutrophil, 25(OH)D3, Duration of T2DM, HbA1c and ApoA1. The model not only demonstrated excellent predictive ability, but also filled an important gap in previous studies through external validation. During the development and external validation phases of our study, the model achieved AUC values of 0.834 and 0.851, respectively, which is a significant advantage over previous models (0.709 and 704, respectively)^20^. The calibration curve and Hosmer–Lemeshow (H–L) test results further confirmed the high accuracy of the model in adjusting the actual and predicted probabilities of outcome events. In addition, decision curve analysis (DCA) demonstrated that implementation of the model in clinical practice yields significant net benefits, thus highlighting its practical relevance in the field of medical research.

Our study reveals a significant positive correlation between ApoA1 and the severity of DR. Previous studies addressing the effect of ApoA1 on DR are scarce. However, recent studies have shown a positive correlation between high-density lipoprotein (HDL) and DR. Given that ApoA1 is the major structural protein of HDL, this finding provides some support for the hypothesis that ApoA1 may have an effect on DR. In a pivotal case–control study, Simo et al.^21^ compared ApoA1 expression in the retinas of diabetic and nondiabetic patients. It was found that overexpression of ApoA1 is an early phenomenon in the retinas of diabetic patients, which may be closely related to the pathophysiologic process of DR. In contrast, the study by Vinodhini et al.^22^ did not manage to establish a direct link between ApoA1 levels and DR severity. The widespread use of statins in the study and control groups may have contributed to this finding. In another study, Ankit and his team^23^ found that diabetic patients had higher levels of ApoA1 in vitreous fluid and retinal pigment epithelial cells compared to nondiabetic patients, which may increase the risk of developing diabetes. ApoA1 has an antifibrinolytic role in the atherosclerotic process and the plasma fibrinolytic system, and high levels of ApoA1 may lead to small-vessel occlusion^24^, which could promote the development of DR, which is consistent with the results of our study.

In addition, we noted differences in the composition of ApoE and HDL. Apolipoproteins are not only structural proteins, but also have enzymatic activity and receptor binding. The cholesterol in HDL, on the other hand, actually refers only to the cholesterol lipid content in it. Past studies have often focused only on HDL and ignored the role of other important lipoproteins, which may have led to less comprehensive and rigorous scientific studies. Furthermore, the metabolic environment of diabetic patients is significantly different from that of healthy individuals. In the setting of diabetes, lipoproteins undergo non-enzymatic glycation, oxidation, and modification of advanced glycation end products, and these changes may have an impact on test results. In addition, the accuracy of lipoprotein measurements may be variable, with sample handling, non-enzymatic glycation and oxidation having varying effects on different assays.

It is worth mentioning that the level of ApoA1 is more stable than the level of blood lipids, especially in the group of diabetes patients, and its level will not be significantly affected by the pre meal state. Therefore, compared with high-density lipoprotein and lipid indicators, apolipoprotein A1 may be a more accurate and reliable biomarker of diabetes retinopathy (DR).

Vitamin D, a fat soluble vitamin, exists in various forms in the human body. Among them, serum 25 hydroxyvitamin D3 [25(OH)D3] is its main circulating form and is widely considered a key indicator for evaluating vitamin D levels. In recent years, the relationship between vitamin D and microvascular complications of type 2 diabetes has gradually received extensive attention from scholars at home and abroad. Among them, diabetes retinopathy (DR), as one of the major microvascular complications of diabetes, is particularly worth studying. In a meta-analysis of 17,664 type 2 diabetes patients^12^, the study found that the risk of DR was significantly increased in patients with vitamin D deficiency. In a cross-sectional study, Millen et al.^25^ divided 15,792 subjects into three groups based on their serum 25(OH)D3 levels: vitamin D sufficient, vitamin D insufficient, and vitamin D deficient. The results showed that the incidence of DR was higher in vitamin D deficient subjects, consistent with the results of this study, further demonstrating the correlation between serum vitamin D status and DR risk. This suggests that 25(OH)D3 may serve as an important serum marker for predicting DR, which can aid in early screening of DR patients. In addition, Jamali et al.^14^ found in vitro experiments on rats that mice carrying vitamin D receptors performed better in promoting pericellular density arrest and endothelial cell survival compared to mice without vitamin D receptors, and were able to inhibit ischemia mediated retinal neovascularization through vitamin D. Lazzara et al.^13^ also found in vitro experiments on human cells that supplementing vitamin D can rebuild the integrity of the blood retinal internal barrier by increasing the expression of VE cadherin and ZO-1 proteins, protecting human retinal endothelial cells from high glucose induced damage. These in vitro studies provide new insights into the role of vitamin D in retinal protection.

Therefore, in the future health management of DR patients, attention should be paid to monitoring serum 25(OH)D3, and appropriate supplementation of vitamin D should be considered as one of the possible treatment strategies for preventing or improving DR.

Diabetes duration is an important independent risk factor in DR prediction models and is recognized as a major diabetic complication risk factor. Diabetes duration is strongly associated with the development of a variety of diabetes-related macrovascular and microvascular complications. Liu YY et al.^26^ in a 16-year cohort study of patients with T2DM, found that the prevalence of DR was 25% at 10 years of disease duration, and increased dramatically to 50% at 15 years of disease duration. Joanne et al.^27^ also found that DR was a significant risk factor in the prediction model of DR, and was recognized as a major risk factor for diabetic complications. A pooled analysis of 35 epidemiologic studies also found that the prevalence of DR increased significantly with disease duration, ranging from 21.1% in patients with a disease duration of less than 10 years to 76.3% when the disease duration was more than 20 years. Al Rubeaan et al.^28^ showed that diabetes with a disease duration of more than 10 years and older age of the patient were significant risk factors for DR. There was a 50-fold increase in the prevalence of DR between the youngest group with the shortest Duration of T2DM and the oldest group with the longest duration^27^. A study by Alramadan et al.^29^ also found that increasing age was significantly associated with the onset of DR. The prevalence of DR in patients ≤ 60, 61–70 and ≥ 71 years of age was 38.5%, 46.8% and 55.4%, respectively, and these patients generally had a long duration of type 2 diabetes. The baseline analysis of this study showed that the Duration of T2DM in DR patients was significantly longer than that in non-DR patients, and the risk of DR increased significantly with longer disease duration, which is consistent with national and international literature. Therefore, the frequency of screening should be increased in patients with longer disease duration in order to prevent or slow down the development of DR at an early stage.

It is speculated that the Duration of T2DM may be an indicator reflecting the overall level of blood glucose control and the exposure period of risk factors. Calderon et al.^30^ and Solomon SD et al.^31^ believe that blood glucose indicators are closely related to the severity of diabetes retinopathy (DR). For patients with T2DM, the higher the level of HbA1C, the higher the incidence of DR; On the contrary, when the HbA1C level decreases, the risk of DR occurrence will also decrease accordingly. In a cross-sectional study^32^ involving about 127,000 diabetes patients in northern Jiangsu, China, high HbA1C levels were confirmed to be an important risk factor for DR.

In this study, the data in Table 1 clearly shows that the levels of HbA1C in the DR patient population are significantly higher than those in the non DR patient population, and there is a statistically significant difference between the two. This further confirms the role of HbA1C as an independent risk factor for DR patients. HbA1C is a product formed by the combination of blood glucose and hemoglobin, usually used to reflect the patient's blood glucose control status over the past 8 to 12 weeks. Although the production process of glycosylated hemoglobin is relatively slow, it is not easy to decompose once it is formed, so it becomes an important indicator to monitor the blood sugar level of diabetes patients. Long term hyperglycemia may trigger irreversible vascular changes, but glycated hemoglobin, as an important indicator of blood glucose levels, is of great importance^33^. The level of glycosylated hemoglobin not only reflects the blood sugar content, but also is closely related to the complications related to diabetes, such as microvascular and macrovascular diseases^34^. Recent studies^35^ have shown that as HbA1c levels increase, it may affect the ability of hemoglobin to carry oxygen, thereby affecting retinal function, while a decrease in HbA1c levels helps to reduce the risk of DR^30^. Therefore, for patients with type 2 diabetes, strict blood glucose control and reduction of HbA1c level are still the key to diabetes management and prevention or mitigation of DR disease progress.

In the present study, we establish for the first time neutrophils as a protective factor in DR, a finding that has rarely been mentioned in previous studies. As the most abundant leukocyte type in the human body, accounting for up to 65% of all neutrophils, neutrophils, in addition to their well-known phagocytosis, have a unique mechanism of suicidal attack, neutrophil net trapping death (NETosis). This process culminates in the formation of neutrophil extracellular traps (NETs)^36^, and a large number of recent studies^37–39^ have demonstrated that NETs play an important role in the development and progression of DR. This is mainly because the hyperglycemic environment stimulates neutrophils to produce superoxide and a variety of cytokines that promote NETosis.

In an experimental study^40^, researchers found that serum levels of tumor necrosis factor-alpha (TNF-α) were elevated in diabetic patients, a change that stimulated the formation of NETs in neutrophils and led to the release of intracellular serine proteases, including neutrophil elastase (NE), which, in turn, led to an increase in the levels of NE and enzyme activity detected in the serum of diabetic patients. In turn, the increase in NE promotes the aggregation of neutrophils toward sites of inflammation and creates a negative feedback regulatory mechanism, which may further exacerbate the development of autoimmune diabetes. Thus, in diabetic patients, a decrease in the number of neutrophils and an increase in NETs are clearly key factors in this complex process. During the formation of NETs, neutrophils release negatively charged free DNA that activates coagulation factor XII, which triggers a series of reactions, including activation of kinin-releasing enzyme and production of bradykinin. Bradykinin binds to G protein-coupled bradykinin B1 and B2 receptors, which are highly expressed in the retina, and thus participates in both acute and chronic inflammatory injury processes in the DR^41^. Neutrophils, which originate mainly from the bone marrow and are released into the peripheral blood^42^, play an important role in innate immunity, acting as the first line of cellular defense. Therefore, a decrease in neutrophil numbers may signal an increase in NETs in the body, thereby promoting the onset and progression of DR, which provides an important early warning signal for diabetic patients. Few current predictive models involve neutrophils, making this study innovative in its exploration of this area.

In this study, an innovative predictive model was developed for assessing the risk of DR based on the analysis of risk factors associated with DR. Through external validation, the model showed excellent diagnostic efficacy and goodness of fit, providing an effective tool for early prediction of DR. Considering the large population of diabetic patients and the uneven distribution of healthcare resources in China, especially in remote areas, screening for DR is extremely challenging. The novel prediction model developed in this study, based on existing research, will greatly improve the current screening status quo and provide an efficient and practical tool. By utilizing simple and easily accessible data for the initial assessment of diabetic patients, this model not only provides a scientific assessment basis for primary and general practitioners, but also helps to guide further diagnostic and therapeutic decisions, thus effectively reducing the incidence of DR. In addition, despite the results of this study, as a retrospective study, its conclusions still need to be validated and strengthened by future prospective studies. This not only provides a more reliable clinical basis for the prevention and treatment of DR, but also points the way for future research directions.

To summarize, this predictive model serves as an effective tool for assessing the risk of DR, incorporating five key clinical features. It is not only applicable to areas with uneven distribution of healthcare resources, but can also play an important role in primary care settings where fundus examination is not available. The application of this model will help clinicians to intervene at an early stage, thus effectively reducing the morbidity and mortality of DR in the future, which will have far-reaching implications for improving the long-term health prognosis of diabetic patients.

### Supplementary Information

Supplementary Table S1.

## Data Availability

The data underlying this article will be shared on reasonable request to the corresponding author.

## References

[1] Lovic, D. *et al.* The growing epidemic of diabetes mellitus. *Curr. Vasc. Pharmacol.* **18**(2), 104–109. 10.2174/1570161117666190405165911 (2020). 30961501 10.2174/1570161117666190405165911

[2] IDF Diabetes Atlas. https://diabetesatlas.org/atlas/ninth-edition (Accessed 27 Mar 2023).

[3] Abramoff, M. D. *et al.* Approach for a clinically useful comprehensive classification of vascular and neural aspects of diabetic retinal disease. *Investig. Ophthalmol. Vis. Sci.* **59**(1), 519–527. 10.1167/iovs.17-21873 (2018). 29372250 10.1167/iovs.17-21873 PMC5786342

[4] American Diabetes Association Professional Practice Committee. 12. Retinopathy, neuropathy, and foot care: Standards of medical care in diabetes—2022. *Diabetes Care* **45**(1), S185–S194. 10.2337/dc22-S012 (2022). 34964887 10.2337/dc22-S012

[5] Li, Y. *et al.* Nomogram for prediction of diabetic retinopathy among type 2 diabetes population in Xinjiang, China. *Diabetes Metab. Syndr. Obes.* **7**(15), 1077–1089. 10.2147/DMSO.S354611 (2022). 10.2147/DMSO.S354611 PMC8999722 35418766

[6] Jia, W. *et al.* Standards of medical care for type 2 diabetes in China 2019. *Diabetes/Metab. Res. Rev.* **35**(6), e3158. 10.1002/dmrr.3158 (2019). 30908791 10.1002/dmrr.3158

[7] Antonetti, D., Silva, P. & Stitt, A. Current understanding of the molecular and cellular pathology of diabetic retinopathy. *Nat. Rev. Endocrinol.* **17**(4), 195–206. 10.1038/s41575-021-00467-9 (2021). 33469209 10.1038/s41575-021-00467-9 PMC9053333

[8] Zhang, Y. *et al.* Artificial intelligence-enabled screening for diabetic retinopathy: A real-world, multicenter and prospective study. *BMJ Open Diabetes Res. Care* **8**(1), e001596. 10.1136/bmjdrc-2020-001596 (2020). 33087340 10.1136/bmjdrc-2020-001596 PMC7580048

[9] Yang, Y., Tan, J. & He, Y. Predictive model for diabetic retinopathy under limited medical resources: A multicenter diagnostic study. *Front. Endocrinol.* **13**, 1664–2392. 10.3389/fendo.2022.1099302 (2023). 10.3389/fendo.2022.1099302 PMC9849672 36686423

[10] Yadav, P. *et al.* Impact of severity of diabetic retinopathy on quality of life in type 2 Indian diabetic patients. *Int. J. Community Med. Public Health* **8**(1), 207. 10.18203/2394-6040.ijcmph20205694 (2020). 10.18203/2394-6040.ijcmph20205694

[11] Li, Y. *et al.* Training and external validation of a predict nomogram for type 2 diabetic peripheral neuropathy. *Diagnostics* **13**, 1265. 10.3390/diagnostics13071265 (2023). 37046484 10.3390/diagnostics13071265 PMC10093299

[12] Luo, B., Gao, F. & Qin, L. The association between vitamin d deficiency and diabetic retinopathy in type 2 diabetes: A meta-analysis of observational studies. *Nutrients* **9**(3), 307. 10.3390/nu9030307 (2017). 28335514 10.3390/nu9030307 PMC5372970

[13] Lazzara, F. *et al.* Vitamin D3 preserves blood retinal barrier integrity in an in vitro model of diabetic retinopathy. *Front. Pharmacol.* **13**, 971164. 10.3389/fphar.2022.971164 (2022). 36091806 10.3389/fphar.2022.971164 PMC9458952

[14] Jamali, N. *et al.* Vitamin D receptor expression is essential during retinal vascular development and attenuation of neovascularization by 1, 25(OH)2D3. *PLoS ONE* **12**(12), e0190131. 10.1371/journal.pone.0190131 (2017). 29272316 10.1371/journal.pone.0190131 PMC5741250

[15] Chinese Diabetes Society. Guideline for the prevention and treatment of type 2 diabetes mellitus in China (2017 edition). *Chin. J. Diabetes Mellitus* **10**(1), 4–67. 10.3760/cma.j.issn.1674-5809.2018.01.003 (2018). 10.3760/cma.j.issn.1674-5809.2018.01.003

[16] Tibshirani, R. Regression shrinkage and selection via the lasso. *J. R. Stat. Soc. Ser. B*10.1111/j.2517-6161.1996.tb02080.x (1996). 10.1111/j.2517-6161.1996.tb02080.x

[17] Hanley, J. & Mcneil, B. A method of comparing the areas under receiver operating characteristic curves derived from the same cases. *Radiology* **148**(3), 839–843. 10.1148/radiology.148.3.6878708 (1983). 6878708 10.1148/radiology.148.3.6878708

[18] Paul, P., Pennell, M. L. & Lemeshow, S. Standardizing the power of the Hosmer-Lemeshow goodness of fit test in large data sets. *Stat. Med.*10.1002/sim.5525 (2013). 22833304 10.1002/sim.5525

[19] Fitzgerald, M., Saville, B. R. & Lewis, R. J. Decision curve analysis. *JAMA.* **313**, 409–410. 10.1001/jama.2015.37 (2015). 25626037 10.1001/jama.2015.37

[20] Wang, G. *et al.* Development and validation of a diabetic retinopathy risk prediction model for middle-aged patients with type 2 diabetes mellitus. *Front. Endocrinol.* **14**, 1132036. 10.3389/fendo.2023.1132036 (2023). 10.3389/fendo.2023.1132036 PMC10050549 37008912

[21] Simó, R. *et al.* Apolipoprotein A1 is overexpressed in the retina of diabetic patients. *Am. J. Ophthalmol.* **147**, 319–25.e1. 10.1016/j.ajo.2008.08.009 (2009). 18848320 10.1016/j.ajo.2008.08.009

[22] Vinodhini, V. M. *et al.* A study on the pattern of lipid profile and apolipoproteins in patients with diabetic retinopathy. *Int. J. Pharm. Clin. Res.* **5**(1), 1–3 (2013).

[23] Ankit, B. *et al.* Stronger relationship of serum apolipoprotein A-1 and B with diabetic retinopathy than traditional lipids. *Indian J. Endocrinol. Metab.* **21**(1), 102–105. 10.4103/2230-8210.196030 (2017). 28217507 10.4103/2230-8210.196030 PMC5240048

[24] Maioli, M. *et al.* Raised serum apolipoprotein(a) in active diabetic retinopathy. *Diabetologia* **36**(1), 8890. 10.1007/BF00399100 (1993). 10.1007/BF00399100 8436260

[25] Millen, A. E. *et al.* Adequate vitamin D status is associated with the reduced odds of prevalent diabetic retinopathy in African Americans and Caucasians. *Cardiovasc. Diabetol.* **15**(1), 128. 10.1186/s12933-016-0434-1 (2016). 27586865 10.1186/s12933-016-0434-1 PMC5009647

[26] Liu, Y. Y. *et al.* Glycemic exposure and blood pressure influencing progression and remission of diabetic retinopathy: A longitudinal cohort study in GoDARTS. *Diabetes Care* **36**(12), 3979–3984. 10.2337/dc12-2392 (2013). 24170761 10.2337/dc12-2392 PMC3836116

[27] Ding, J. & Wong, T. Y. Current epidemiology of diabetic retinopathy and diabetic macular edema. *Curr. Diabetes Rep.* **12**(4), 346–354. 10.1007/s11892-012-0283-6 (2012). 10.1007/s11892-012-0283-6 22585044

[28] Al-Rubeaan, K. *et al.* Diabetic retinopathy and its risk factors in a society with a type 2 diabetes epidemic: A Saudi National Diabetes Registry-based study. *Acta Ophthalmol.* **93**, e140–e147. 10.1111/aos.12532 (2015). 25270515 10.1111/aos.12532

[29] Alramadan, M. J. *et al.* Lifestyle factors and macro- and micro-vascular complications among people with type 2 diabetes in Saudi Arabia. *Diabetes Metab. Syndr. Clin. Res. Rev.* **13**(1), 484–491. 10.1016/j.dsx.2018.11.007 (2019). 10.1016/j.dsx.2018.11.007 30641750

[30] Calderon, G. D. *et al.* Oxidative stress and diabetic retinopathy: Development and treatment. *Eye* **31**(8), 1122–1130. 10.1038/eye.2017.64 (2017). 28452994 10.1038/eye.2017.64 PMC5558229

[31] Solomon, S. D. & Goldberg, M. F. ETDRS grading of diabetic retinopathy: Still the gold standard?. *Ophthalmic Res.* **62**(4), 190–195. 10.1159/000501372 (2019). 31454808 10.1159/000501372

[32] Feng, R. *et al.* Diabetes onset at an earlier age and high HbA1c levels as risk factors of diabetic retinopathy. *Int. J. Ophthalmol.* **14**(2), 269–276. 10.18240/IJO.2021.02.14 (2021). 33614457 10.18240/IJO.2021.02.14 PMC7840379

[33] Catalani, E. & Cervia, D. Diabetic retinopathy: A matter of retinal ganglion cell homeostasis. *Neural Regener. Res.* **15**(7), 1253–1254. 10.4103/1673-5374.272577 (2020). 10.4103/1673-5374.272577 PMC7047784 31960808

[34] Wang, S. Y. *et al.* Incidence and risk factors for developing diabetic retinopathy among youths with Type 1 or Type 2 diabetes throughout the United States. *Ophthalmology* **124**(4), 424–430. 10.1016/j.ophtha.2016.10.031 (2017). 27914837 10.1016/j.ophtha.2016.10.031 PMC5728116

[35] Jorgensen, C. M., Hardarson, S. H. & Bek, T. The oxygen saturation in retinal vessels from diabetic patients depends on the severity and type of visionthreatening retinopathy. *Acta Ophthalmol.* **92**(1), 34–39. 10.1111/aos.12283 (2014). 24330421 10.1111/aos.12283

[36] Chu, Z. Q. *et al.* Neutrophil extracellular traps in gastrointestinal cancer. *World J. Gastroenterol.* **27**(33), 5474–5487. 10.3748/wjg.v27.i33.5474 (2021). 34588746 10.3748/wjg.v27.i33.5474 PMC8433615

[37] Liu, H. *et al.* Neutrophil elastase contributes to the pathological vascular permeability characteristic of diabetic retinopathy. *Diabetologia.* **62**(12), 2365–2374. 10.1007/s00125-019-04998-4 (2019). 31612267 10.1007/s00125-019-04998-4 PMC6866660

[38] Song, D. Y. *et al.* Activation of factor XII and Kallikrein-Kinin system combined with neutrophil extracellular trap formation in diabetic retinopathy. *Exp. Clin. Endocrinol. Diabetes.* **129**(8), 560–565. 10.1055/a-0981-6023 (2021). 31426112 10.1055/a-0981-6023

[39] Binet, F. *et al.* Neutrophil extracellular traps target senescent vasculature for tissue remodeling in retinopathy. *Science.* **369**(6506), eaay5356. 10.1126/science.aay5356 (2020). 32820093 10.1126/science.aay5356

[40] Wang, Y., Xiao, Y. & Zhong, L. Increased neutrophil elastase and proteinase 3 and augmented NETosis are closely associated with β-cell autoimmunity in patients with type 1 diabetes. *Diabetes.* **63**(12), 4239–4248. 10.2337/db14-0480 (2014). 25092677 10.2337/db14-0480

[41] Sainz, I. M., Pixley, R. A. & Colman, R. W. Fifty years of research on the plasma Kallikrein-Kinin system: From protein structure and function to cell biology and in-vivo pathophysiology. *Thromb. Haemost.* **98**(1), 77–83. 10.1160/Th07-04-0250 (2007). 17597995 10.1160/Th07-04-0250

[42] Wang, L. *et al.* Hyperglycemia induces neutrophil extracellular traps formation through an NADPH oxidase-dependent pathway in diabetic retinopathy. *Front. Immunol.* **8**(9), 3076. 10.3389/fimmu.2018.03076 (2019). 10.3389/fimmu.2018.03076 PMC6331470 30671057

