# Supplementary material for: Development and external validation of a predictive model for type 2 diabetic retinopathy
Source: Sci Rep. 2024 Jul 20;14:16741. doi: 10.1038/s41598-024-67533-5 (PMC11271465; doi:10.1038/s41598-024-67533-5)
Supplement: Supplementary file 1 — Supplementary Table S1. [file 41598_2024_67533_MOESM1_ESM.docx]

**Supporting information**

**Table S1: Net benefits for different threshold probabilities in the external validation cohort**

| Threshold | All | None | Predmodel |
| --- | --- | --- | --- |
| 0.01 | 0.487599488 | 0 | 0.487599488 |
| 0.02 | 0.482370911 | 0 | 0.482370911 |
| 0.03 | 0.477034529 | 0 | 0.477034529 |
| 0.04 | 0.471586972 | 0 | 0.471673597 |
| 0.05 | 0.466024729 | 0 | 0.466243572 |
| 0.06 | 0.460344141 | 0 | 0.461538462 |
| 0.07 | 0.45454139 | 0 | 0.454966132 |
| 0.08 | 0.448612492 | 0 | 0.449064449 |
| 0.09 | 0.442553289 | 0 | 0.442998789 |
| 0.10 | 0.436359436 | 0 | 0.439015939 |
| 0.11 | 0.430026396 | 0 | 0.435177182 |
| 0.12 | 0.423549424 | 0 | 0.427423927 |
| 0.13 | 0.416923555 | 0 | 0.421344421 |
| 0.14 | 0.410143596 | 0 | 0.417009138 |
| 0.15 | 0.403204109 | 0 | 0.404916228 |
| 0.16 | 0.396099396 | 0 | 0.397683398 |
| 0.17 | 0.388823485 | 0 | 0.394158756 |
| 0.18 | 0.381370113 | 0 | 0.391638355 |
| 0.19 | 0.373732707 | 0 | 0.391814892 |
| 0.20 | 0.365904366 | 0 | 0.388513514 |
| 0.21 | 0.357877839 | 0 | 0.385352246 |
| 0.22 | 0.349645503 | 0 | 0.38107042 |
| 0.23 | 0.341199341 | 0 | 0.37967438 |
| 0.24 | 0.332530911 | 0 | 0.37761243 |
| 0.25 | 0.323631324 | 0 | 0.376645877 |
| 0.26 | 0.314491206 | 0 | 0.372422318 |
| 0.27 | 0.305100675 | 0 | 0.368495999 |
| 0.28 | 0.295449295 | 0 | 0.362612613 |
| 0.29 | 0.285526046 | 0 | 0.356797751 |
| 0.30 | 0.275319275 | 0 | 0.355954856 |
| 0.31 | 0.264816656 | 0 | 0.351517069 |
| 0.32 | 0.254005136 | 0 | 0.349516938 |
| 0.33 | 0.242870885 | 0 | 0.347239892 |
| 0.34 | 0.231399231 | 0 | 0.342405342 |
| 0.35 | 0.219574604 | 0 | 0.340236686 |
| 0.36 | 0.207380457 | 0 | 0.338617464 |
| 0.37 | 0.194799195 | 0 | 0.337656338 |
| 0.38 | 0.181812085 | 0 | 0.335356448 |
| 0.39 | 0.168399168 | 0 | 0.33122593 |
| 0.40 | 0.154539155 | 0 | 0.328482328 |
| 0.41 | 0.14020931 | 0 | 0.324095282 |
| 0.42 | 0.125385332 | 0 | 0.323643272 |
| 0.43 | 0.110041215 | 0 | 0.319418609 |
| 0.44 | 0.094149094 | 0 | 0.316528067 |
| 0.45 | 0.077679078 | 0 | 0.316386316 |
| 0.46 | 0.060599061 | 0 | 0.311503812 |
| 0.47 | 0.042874515 | 0 | 0.31159534 |
| 0.48 | 0.024468255 | 0 | 0.31177035 |
| 0.49 | 0.005340182 | 0 | 0.307203131 |
| 0.50 | -0.014553015 | 0 | 0.305613306 |
| 0.51 | -0.035258178 | 0 | 0.304170733 |
| 0.52 | -0.056826057 | 0 | 0.301715177 |
| 0.53 | -0.079311718 | 0 | 0.297076127 |
| 0.54 | -0.102775016 | 0 | 0.294811534 |
| 0.55 | -0.127281127 | 0 | 0.293716794 |
| 0.56 | -0.152901153 | 0 | 0.292761293 |
| 0.57 | -0.179712808 | 0 | 0.290915244 |
| 0.58 | -0.207801208 | 0 | 0.286753787 |
| 0.59 | -0.237259774 | 0 | 0.285051468 |
| 0.60 | -0.268191268 | 0 | 0.282744283 |
| 0.61 | -0.300708993 | 0 | 0.27322885 |
| 0.62 | -0.334938177 | 0 | 0.26742532 |
| 0.63 | -0.371017587 | 0 | 0.267629376 |
| 0.64 | -0.409101409 | 0 | 0.258373758 |
| 0.65 | -0.449361449 | 0 | 0.245767746 |
| 0.66 | -0.491989727 | 0 | 0.247156659 |
| 0.67 | -0.537201537 | 0 | 0.23977824 |
| 0.68 | -0.585239085 | 0 | 0.233627859 |
| 0.69 | -0.63637583 | 0 | 0.237911609 |
| 0.70 | -0.690921691 | 0 | 0.23042273 |
| 0.71 | -0.749229335 | 0 | 0.225786795 |
| 0.72 | -0.811701812 | 0 | 0.222750223 |
| 0.73 | -0.878801879 | 0 | 0.218911219 |
| 0.74 | -0.95106349 | 0 | 0.210299056 |
| 0.75 | -1.029106029 | 0 | 0.196465696 |
| 0.76 | -1.113652114 | 0 | 0.187110187 |
| 0.77 | -1.205550032 | 0 | 0.178794179 |
| 0.78 | -1.305802306 | 0 | 0.16953317 |
| 0.79 | -1.415602416 | 0 | 0.156469656 |
| 0.80 | -1.536382536 | 0 | 0.144490644 |
| 0.81 | -1.669876354 | 0 | 0.134916293 |
| 0.82 | -1.818202818 | 0 | 0.128667129 |
| 0.83 | -1.983979455 | 0 | 0.111776935 |
| 0.84 | -2.17047817 | 0 | 0.109407484 |
| 0.85 | -2.381843382 | 0 | 0.097020097 |
| 0.86 | -2.623403623 | 0 | 0.07959608 |
| 0.87 | -2.902126979 | 0 | 0.08052135 |
| 0.88 | -3.227304227 | 0 | 0.065835066 |
| 0.89 | -3.611604612 | 0 | 0.064921565 |
| 0.90 | -4.072765073 | 0 | 0.071725572 |
| 0.91 | -4.636405636 | 0 | 0.062139062 |
| 0.92 | -5.340956341 | 0 | 0.055613306 |
| 0.93 | -6.246807247 | 0 | 0.035046035 |
| 0.94 | -7.454608455 | 0 | 0.02009702 |
| 0.95 | -9.145530146 | 0 | 0.003118503 |
| 0.96 | -11.68191268 | 0 | -0.015592516 |
| 0.97 | -15.90921691 | 0 | -0.027373527 |
| 0.98 | -24.36382536 | 0 | 0.002079002 |
| 0.99 | -49.72765073 | 0 | 0 |
